# Protease Inhibition—An Established Strategy to Combat Infectious Diseases

**DOI:** 10.3390/ijms22115762

**Published:** 2021-05-28

**Authors:** Daniel Sojka, Pavla Šnebergerová, Luïse Robbertse

**Affiliations:** 1 Biology Centre, Institute of Parasitology, Academy of Sciences of the Czech Republic, Branišovská 1160/31, CZ-37005 České Budějovice, Czech Republic; snebep00@prf.jcu.cz (P.Š.); luise.robbertse@paru.cas.cz (L.R.); 2 Faculty of Science, University of South Bohemia in České Budějovice, Branišovská 1760c, CZ-37005 České Budějovice, Czech Republic

**Keywords:** protease, parasites, inhibition, therapy, infectious diseases

## Abstract

Therapeutic agents with novel mechanisms of action are urgently needed to counter the emergence of drug-resistant infections. Several decades of research into proteases of disease agents have revealed enzymes well suited for target-based drug development. Among them are the three recently validated proteolytic targets: proteasomes of the malarial parasite *Plasmodium falciparum,* aspartyl proteases of *P. falciparum* (plasmepsins) and the Sars-CoV-2 viral proteases. Despite some unfulfilled expectations over previous decades, the three reviewed targets clearly demonstrate that selective protease inhibitors provide effective therapeutic solutions for the two most impacting infectious diseases nowadays—malaria and COVID-19.

## 1. Introduction

Infectious diseases, along with starvation, limited water resources and the lack of shelter, are among the main factors threatening the health and prosperity of the world’s growing human population. Significant proportions of infectious diseases are caused by parasites [1], the most common human infections being toxoplasmosis, ascariasis, ancylostomiasis and trichomoniasis. Although relatively less common, malaria, amebiasis, leishmaniasis, schistosomiasis, sleeping sickness and Chagas disease are dangerous infections that affect millions of individuals worldwide and thus represent a huge social and economic burden [2]. Livestock and wildlife infections are also of great importance due to their impact on global economies and prosperity of the human population. Some of the animal important harmful infectious agents, such as the apicomplexan parasites of the genus *Babesia,* vectored by *Ixodes* ticks, can be passed from animals to people and cause zoonotic diseases (zoonoses) [3].

Proteolysis is the breakdown of proteins via enzymatic hydrolysis of peptide bonds linking their amino acid chains. Uncatalyzed proteolysis is extremely slow, which is why living organisms have developed enzymes catalyzing peptide bond hydrolysis. These enzymes, collectively referred to as proteases, have been present since the beginning of evolution [4]. Proteases play key roles in almost every biological phenomenon inside and outside individual organisms. To their typical roles belongs the activation of other enzymes by their targeted processing, leading to the creation of active sites accessible to substrates or, conversely, the inactivation of proteins by their proteolytic degradation [4]. Proteases are divided into seven groups [5]. The majority of them are represented by metallo-, serine, aspartyl, cysteine and threonine proteases. Metalloproteases are those that have a divalent metal ion bound to residues at a catalytic site, while others are classified with respect to catalytic amino acid residues in the active site. Serine proteolytic enzymes are the most abundant in nature, followed by metallo-, cysteine, aspartate and threonine proteases, respectively [6].

## 2. Protease Inhibition as a Strategy for the Treatment of Infectious Diseases

Targeting proteases with low-molecular-weight inhibitors is a valid therapeutic approach and some protease inhibitors have been developed into highly successful drugs for various diseases including hypertension, diabetes and specific types of cancer [7]. Proteolytic enzymes are also valuable targets for the development of novel drugs for infectious diseases because they belong to major virulent factors of infectious agents with important roles in their development, reproduction and interactions with host/invertebrate vector tissues [8,9]. Evidence that targeted inhibition of proteases produced by disease agents can, relatively quickly—compar to antibiotics, stop or eliminate infection dates back to the end of the last century. At that time (1995), retroviral HIV protease inhibitors (HIV-PIs), including Saquinavir [10], Lopinavir and Ritonavir [11], displayed their potential to interfere with virus reproduction and were approved by the US Food and Drug Administration (FDA) for therapeutic intervention against HIV infection. The mechanism of action of HIV-PIs involves selective blocking of the retroviral protease, resulting in disabled processing of the long polypeptide encoded by the RNA genome of the virus into constituent viral proteins. Inhibiting the activity of the protease is therefore an attractive means to prevent mature virion production [12]. Since then, new inhibitors and their combinations have appeared. Nowadays there are ten FDA-approved HIV-PIs on the market and they are key components of HIV antiretroviral therapy (ART), transforming this deadly disease into a more manageable chronic infection. Thus, despite the observed adverse effects (discussed in Section 6), HIV-PIs remain the clearest success of protease inhibition-based therapy [13].

Since the discovery and approval of HIV-PIs, targeted protease inhibition by low-molecular-weight compounds is considered to be a potential strategy against parasite infections. The papain-like cysteine protease cruzain (aka cruzipain) has been shown to be essential for the viability and virulence of *Trypanosoma cruzi,* causing Chagas disease. Studies with vinyl-sulfone, an irreversible inhibitor of cruzain K777 (aka K11777), displayed its effectiveness in preclinical models of *T. cruzi* infection, including immunocompetent and immunodeficient mice and dogs, and has been shown to be effective against other parasitic infections including schistosomiasis, hookworm infections and cryptosporidiosis [14]. 

In the following years, inhibition of proteolytic enzymes fell far short of initial expectations as a therapeutic strategy [7]. This resulted in certain skepticism and deviation from this approach, e.g., K777 encountered problems in tolerability during clinical trials, leading to non-approval as a commercial drug [14]. This was reflected in lowered interest in protease-based drug discovery. “Rational design” of protease inhibitors was altered with fashionable approaches based on combinatorial chemistry and high-throughput screenings as supposedly being more potent ways to discover innovative drugs for infectious diseases. However, the imaginary pendulum has turned back over the last decade and structural biology and protein engineering of active biomolecules is now undoubtedly an advanced science. It has found a stable place in the development of new drugs and selective protease inhibitors are again among the top drug candidates. As clearly described by Dr. Clare Sansom in the November 2009 Chemistry World magazine issue, there are fashions in drug discovery, and they tend to exactly follow the well-known Hype Cycle curve of the US research, consulting and information company Gartner [15]. This curve represents the maturity, acceptance and social application of specific technologies from initial adoption, over exaggerated expectations, subsequent gaps of interest and disillusionment until the establishment of stable productive platforms. This also applies to the “rational design” of new protease inhibitors based on knowledge of the biological role of target enzymes, their high-resolution 3D structures and the physicochemical properties of novel compounds. Evidence is provided by the three following examples of a “renaissance” of this approach. The first two examples are focused on malarial proteases, the third is represented by protease-targeting strategies leading to effective therapeutic solutions against COVID-19.

## 3. Selective Inhibition of Proteasomes 

**Figure 1:**
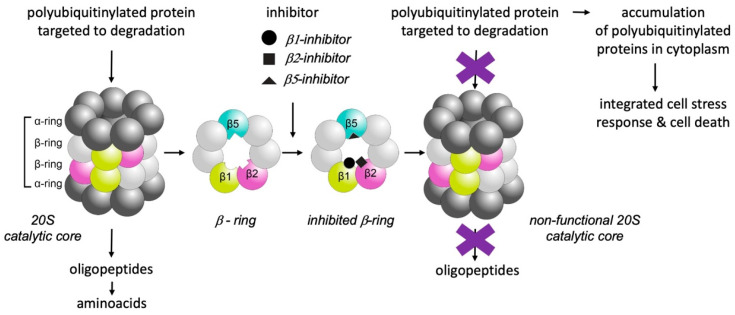
Inhibition of the 20S proteasome catalytic subunits, schematic depiction. The 20S proteasome consists of two outer rings consisting of seven α subunits and two inner rings of seven β subunits. The β1, β2 and β5 subunits are catalytically active and their different substrate specificities are illustrated by the dissimilar shapes of their substrate binding pockets. Proteasome inhibition leads to accumulation of polyubiquitinylated proteins in the cytoplasm and unbalanced protein homeostasis, which causes an integrated stress response and, ultimately, cell death.

Tight regulation of the cellular proteome is critical for normal cellular function, survival and proliferation. Part of the regulatory network is the control of protein synthesis and degradation. In most eukaryotic cells, this control is maintained by the ubiquitin–proteasome system (UPS) [16]. Its final part, the proteasome, is a large protein complex/proteolytic machine responsible for the regulated degradation of poly-ubiquitinylated proteins in the nucleus and cytoplasm of cells [17]. Besides protein homeostasis and the cell stress response, it takes part in the control of various other cellular processes such as cell division [18]. The proteasome proteolytic complex consists of two major parts: the 20S catalytic core particle (720 kDa) and one or two associated 19S regulatory particles (890 kDa). The cylindrical 20S catalytic core of the proteasome is formed by two outer rings of α subunits surrounding two inner rings of seven β subunits, three of which (β1, β2 and β5) are proteases (Figure 1) differing in their specificities including chymotrypsin-like (β5), trypsin-like (β2) and caspase-like (β1) properties. The 19S regulatory cap binds the polyubiquitinylated protein substrate and feeds it to the 20S proteolytic core [18].

Because certain cancer cells are sensitive to proteasome inhibitors, selective proteasome inhibition has become a therapeutic strategy for some types of oncologic disorders. Three proteasome inhibitors—carfilzomib, bortezomib and ixazomib—have been approved for the treatment of multiple myeloma [19]. Moreover, selective inhibition of parasite over host proteasomes has acquired a new reputation as an effective novel strategy for the treatment of infectious diseases including malaria, leishmaniasis, schistosomiasis and Chagas disease [20]. Selective inhibitors are of great importance for the treatment of malaria, as indicated in the first studies testing the proteasome inhibitor effect on *Plasmodium* species [21,22]. It was confirmed that the proteasome plays a crucial role throughout the whole life cycle of the malarial parasite and the inhibitors thus show activity against all life stages of *P. falciparum* [20]. Notably, the effect of proteasome inhibitors on malaria infection might also assist in interrupting the priming of human erythrocytes via exosomes containing functional 20S proteasomes. These modulate the mechanical properties of naïve erythrocytes prior to *P. falciparum* infection during its asexual multiplication in host erythrocytes [23].

Numerous new generations of compounds have been identified since the initial discovery of the selective inhibition of *P. falciparum* by bortezomib and MG-132 [24,25]. Library screening of 670 carfilzomib (epoxyketone) analogues identified PR3 as a potent inhibitor of ring-stage *P. falciparum* replication, being able to reduce parasitic loads in *Plasmodium berghei*-infected mice [26]. An extensive library screening of 1600 proteasome inhibitors identified nine N, C-capped non-covalent peptidyl derivatives, out of which the lead compound had a more than 1450-fold increased selectivity for *P. falciparum* when compared with human foreskin fibroblasts [27]. Screening of a library of boronic-acid-based human proteasome inhibitors led to the identification of four compounds with increased selectivity for *P. falciparum* compared with the mammalian proteasome, with potential for a further increase in their selectivity indexes by chemical modifications [28]. Construction of novel compounds selectively inhibiting the proteasome of malarial parasites is based on the six “established classes” of proteasome inhibitors: β-lactones; α’, β’-epoxyketones, peptide aldehydes; boronic acids; vinyl sulfones and cyclic peptides [29]. To aid in the rational development of potent proteasome inhibitors, in which the parasite proteasome is selectively inhibited over the host proteasome, substrate specificity profiling was used to design the vinyl sulphone-derived compounds WLL-vs, WLW-vs and LLW-vs, based on recently discovered differences in the specificities of the human and *P. falciparum* proteasomes [30]. More recently, asparagine ethylenediamines (AsnEDAs), known as human immunoproteasome inhibitors, were modified for selectivity against the *P. falciparum* proteasome [31]. The selectivity index of newly designed compounds might also be increased by reduced toxicity to the host proteasome [32]. In addition, novel *P. falciparum* proteasome inhibitors act on parasite strains that are resistant to treatment with currently used antimalarials. The effectiveness of various inhibitors against different proteasomal subunits underscores the potential value of treating malaria with combinations of inhibitors in order to minimize the emergence of drug resistance [31]. Protease inhibitors synergize with diverse classes of antimalarial agents, strongly supporting further efforts to develop highly effective complex drugs combating resistant strains of malarial parasites [33].

## 4. Plasmepsins—Rediscovered Molecular Targets for the Treatment of Malaria

Aspartyl protease of *P.* falciparum, plasmepsins (*Plasmodium* pepsins, abbreviated PM), are structurally and evolutionarily related to human pepsin and lysosomal cathepsin D (clan AA, A1 family). These enzymes use two aspartic acid residues in their active site and excel in their ability to specifically find a unique cleavage site within protein bonds to cleave proteins into large peptides. Therefore, they often function as specific endopeptidases performing important biological functions. In Apicomplexan parasites, the cathepsin-D-like aspartyl proteases (ASPs) underwent function-driven evolution by duplication and mutation of the ancestral ASP protease-encoding gene. The subsequent development into six evolutionary subgroups (ASP clades A–F) is associated with various functions important for the parasitic way of life and the complicated course of apicomplexan lifecycles [34,35]. The *P. falciparum* genome encodes ten related ASPs, collectively referred to as plasmepsins (abbreviated as PfPMI-X), with plasmepsin III being tagged as a histo-aspartyl protease (HAP) [36]. Plasmepsins have been considered potential targets of antimalarial drugs after discovering the inhibitory effects of HIV-PIs on malaria [37]. During the invasion of *P. falciparum* host red blood cells, hemoglobin is used as a source of amino acids to meet the parasite’s nutritional requirements for growth and maturation. Therefore, PfPMI, PfPMII, PfPMIV and HAP, located in the digestive vacuole of merozoites, were initially considered to be the enzyme targets of HIV-PIs. However, this was later refuted with reference to considerable functional redundancy of the *P. falciparum* vacuolar digestive system, where plasmepsins and cysteine hemoglobinases (falcipains) act synergistically, greatly reducing the effects of targeted inhibition of digestive plasmepsins [36]. PfPMVI, PfPMVII and PfPMVIII are three plasmepsin isoenzymes that are not produced by the blood stages of *P. falciparum* and are important for the development of malaria in mosquito vectors. The roles of residual plasmepsins PfPMV (ASP clade D), PFPMIX and PfPMX (both ASP clade C) were elucidated for the first time over the last decade. This was due to remarkable progress in designing functional genomic tools for *P. falciparum.* At present, these enzymes represent one of the most promising molecular targets for the development of new antimalarials.

Upon invasion of host erythrocytes, *P. falciparum* produces hundreds of effector proteins that are released to the parasite-surrounding parasitophorous vacuole and beyond into the infected host cell. This is modified to enable intracellular survival and multiplication of the parasite [38]. An important role is played by PfPMV, the endoplasmic reticulum resident ASP of *P. falciparum* [39]. PfPMV is responsible for the proteolytic cleavage of the short RxLxE/Q/D (PEXEL) motif localized at the N-terminus of several hundred parasite-produced proteins [40], enabling their translocation into the host cell via the *Plasmodium* translocon of exported proteins (tagged as PTEX) [41]. PfPMV is thus essential for the development of malaria. Moreover, as it is expressed in both the asexual stages and the gametocytes of *P. falciparum*, PfPMV represents a dual therapeutic as well as transmission-blocking drug target [42]. PfPMV can be effectively inhibited by compounds with a transition-state isostere that mimics the natural PEXEL substrate [43]. The first effective inhibitor, WEHI-916, showed high affinity for the enzyme, but only suboptimal capacity to inhibit growth of *P. falciparum* asexual stages in vitro. The structural properties of WEHI-916 have been further improved by the substitution of P3 arginine with canavanine (cav) [43]. The novel analogue WEHI-842 demonstrated 10-times greater affinity and increased capability to kill the parasite [44]. Thus far, inhibitors with the P3 cav replacement and P2 substitution with either phenylglycine or cyclohexylglycine substituents are considered to be the most potent since they provide 6-times higher efficacy in preventing PEXEL cleavage and killing *P. falciparum* asexual stages than WEHI-842 [45].

**Figure 2:**
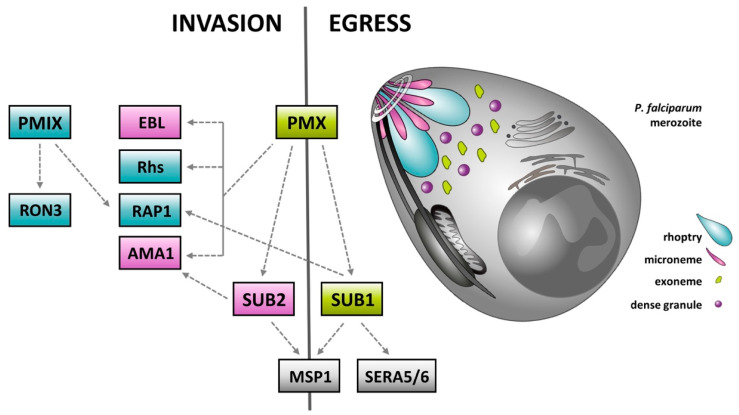
Proteolytic events associated with egress and invasion of red blood cells by *P. falciparum* [47,48,49,50]. Clade C aspartyl protease PMX has been confirmed as the master activating protease of the whole apical complex associated proteolytic system. The central role is maintained by subtilisin-like proteases (SUB1, SUB2), both activated by PMX. SUB1 is released into the parasitophorous vacuole (PV) where it triggers effector proteins such as serine repeat antigens 5 and 6 (SERA5/6)—a pseudoprotease and a cysteine protease regulating parasite egress. Moreover, activity of SERA5/6 and the interactions between SUB1-processed merozoite surface protein 1 (MSP1) and the spectrin network of the erythrocyte cytoskeleton facilitate host erythrocyte rupture during parasite egress. PMX also regulates the process of invasion of naïve erythrocytes by direct or SUB2-mediated processing of merozoite adhesins such as the apical membrane antigen 1 (AMA1). PfPMX-activated SUB2 also contributes to the process of invasion by shedding merozoite surface protein complexes, including MSP1, and sealing of the host erythrocytes upon invasion. Among recently identified substrates of PMX belong erythrocyte binding-like proteins (EBL) and reticulocytes-binding protein homologs (Rhs). They participate in invasion of erythrocytes while initiating changes in the erythrocyte membrane leading to its increased susceptibility to deformation. PMIX is believed to play a role during the invasion by specific processing of several rhoptry proteins. PMIX processes the rhoptry-associated protein 1 (RAP1), which is involved in creation of the parasithophorous vacuole (PV) upon erythrocyte invasion. PMIX also processes the rhoptry neck protein 3 (RON3), that localizes to the membrane of the newly emerging PV and is believed to facilitate import/export of nutrients and effector proteins between the PV and erythrocyte cytoplasm.

Recently, considerable attention has been dedicated to clade C of apicomplexan ASPs comprising the two plasmepsins PfPMIX and PfPMX. These enzymes, analogous to their *Toxoplasma gondii* homologue TgASP3 [46], are associated with the apical complex (AC), a unique apparatus of invasive stages (zoites) of apicomplexan parasites. AC of *P. falciparum* includes a non-secretory structural part consisting of a conoid, polar rings and subpellicular microtubules. These structures are accompanied by several types of secretory organelles, namely filamentous micronemes, claviform rhoptries, dense granules and exonemes. These organelles secrete a vast number of proteins that mediate invasion and egress of host cells and modify both the surface of infected erythrocytes and their surrounding environment. Many of the secreted effector proteins are produced in the form of inactive precursors requiring proteolytic activation by specific proteases. This regulatory mechanism consists of a whole series of proteolytic events that are essential for egress and invasion of host erythrocytes (Figure 2) [47,48,49,50]. 

The central role is maintained by the serine proteases, subtilisins. Subtilisin 1 (SUB1) is involved in merozoite surface remodeling and parasite egress [48]. Subtilisin 2 (SUB2) is a sheddase that releases proteins from the merozoite surface during invasion and that governs erythrocyte membrane sealing upon entry of the parasite [49]. Although the downstream events mediated by these subtilisins are relatively well described, their activation process remained unclear until recently, when PfPMX was confirmed as a master activating protease for both SUB1 and SUB2. Contrary to an earlier hypothesis that PfPMX isoenzyme is involved in microneme and PfPMIX in rhoptry protein processing, the recent findings by Favuzza et al. [50] suggest that these proteases have similar substrate specificities (biochemical selectivity), but factors such as the subcellular localization are important determinants for processing (biological selectivity). Apparently, PfPMX and PfPMIX precursors are activated by autocatalysis and are active along the secretory pathways prior to the apical complex secretory organelles and in a similar way to their *T. gondii* homologue TgASP3 [46].

PfPMIX/X are considered great antimalarial targets, because they can be selectively inhibited by small compounds such as the hydroxy-ethylamine inhibitor 49c [51] or amidohydantoins TCMD-134675, TCMD-136879 and CWHM-117 [52]. Recently, selective PfPMX and dual PfPMIX/X inhibitors were used to demonstrate that PfPMX is the master regulator of invasion and egress. Oral administration of the dual PfPMIX/X inhibitor WM382 cured malaria in murine models, preventing blood infection from the liver and affecting parasite transmission to mosquitoes, indicating multiple roles of clade C ASPs throughout the whole lifecycle of malarial parasites [50].

## 5. Protease Inhibitors as a Potential Therapy for COVID-19 

The third example confirming the establishment of protease-inhibition-based drug development can be found among the recent approaches employed to develop an effective COVID-19 chemotherapy. Coronaviruses, including SARS-CoV-2, contain a genome composed of a long single-stranded RNA molecule that encodes two long polyproteins, pp1a and pp1(a)b. These polyproteins include a complex of proteins for replication/transcription of genetic information in host cells, several structural viral proteins and two proteases: SARS-CoV-2 major protease M^pro^ (also known as 3CL protease or 3CL^pro^) and cysteine papain-like protease PL^pro^ [53]. Both enzymes process the virus-encoded pp1a and pp1(a)b into individual functional protein units. Thus, SARS-CoV-2 proteases play crucial roles in virus replication inside infected cells and naturally represent attractive targets for antiviral drug discovery. Numerous candidate compounds inhibiting M^pro^ have been reported to date. Among them are repurposed drugs designed for other applications as well as de novo designed compounds based on the 3-dimensional structure of M^pro^ (reviewed in [54]). The former are advantageous because of the possibility of rapid entry into further clinical trials, the latter in improved potency and selectivity towards M^pro^. The second protease, PL^pro^, appears even more interesting as a drug target for COVID-19: the papain-like S-CoV-PL^pro^ enzyme of the first severe acute respiratory syndrome coronavirus (SARS-CoV), which emerged in 2002–2003, was found to recognize the tetrapeptide LXGG motif in coronavirus polyproteins. Hydrolysis of peptide bonds on the carboxyl side of glycine at the P1 position leads to the release of nsp1, nsp2 and nsp3 proteins, which are essential for viral replication [55]. In vitro studies determined that in addition to the role in virus replication [56], S-CoV-PL^pro^ activity fundamentally alters the host’s immune responses to viral infection [57,58]. This occurs by proteolytical counteracting the ubiquitinylation and cytokine-induced ISGylation of proteins (labeling of proteins by the product of the interferon-stimulated gene product 15—ISG15). The PL^pro^ protease of the currently emerging coronavirus (S-CoV-2-PL^pro^) possesses a high level of sequence and structural similarity to S-CoV-PL^pro^ and has been characterized for identical roles in virus reproduction and alterations of immune responses [59]. Targeted inhibition of S-CoV-2-PL^pro^ can therefore inhibit its dual role in promoting viral replication and in inhibiting innate immune responses during acute COVID-19 infection. Analogous enzyme characteristics enable immediate application of previous knowledge about SARS-CoV-1 in the search for effective drugs based on the inhibition of S-CoV-2-PL^pro^. Several pieces of work have already described S-CoV-1-PL^pro^ drug-repurposing studies against S-CoV-2-PL^pro^ [56,58,59]. Another tool to rapidly improve selective inhibitors of S-CoV-2-PL^pro^ are protein-substrate and protein-inhibitor 3D structural studies. The first approach helped to explain the specificity of S-CoV-2-PL^pro^ for ISG15 and longer Lys48-linked ubiquitin chains, leading to the identification of inhibitors that show promising antiviral activity in a SARS-CoV-2 infection model [60]. The second approach used protein co-crystal structural analyses that mapped the binding of two synthetic amino-acid-containing inhibitors VIR250 and VIR251 in a complex with S-CoV-2-PL^pro^ [59]. The co-crystal structure of S-CoV-2-PL^pro^ in a complex with GRL0617 indicates that GRL0617 is a non-covalent inhibitor that resides in the ubiquitin-specific protease (USP) domain of S-CoV-2-PL^pro^. Moreover, GRL0167 prevents binding of ISG15 C-terminus to S-CoV-2-PL^pro^ and the binding pocket in S-CoV-2-PL^pro^ contributes disproportionately to the binding energy, which makes it a hot spot for drug discovery [61].

**Figure 3:**
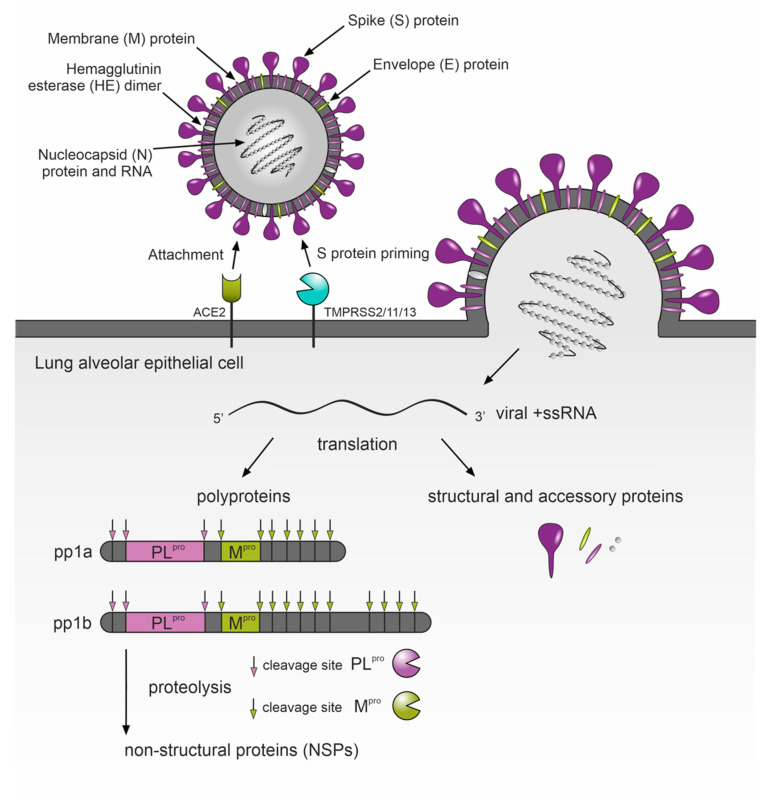
Schematic model of SARS-CoV-2 virus entry into lung cells. The initial attachment of the virus is mediated by SARS-CoV-2 S protein engagement with host ACE2 receptors. Upon ACE2 binding, the viral S protein is proteolytically cleaved by host surface proteases TMPRSS2/11/13 enabling the fusion of the viral envelope and the cell membrane. The nucleocapsid is released into the host cell cytoplasm and the virus initiates replication. The viral +ssRNA encodes two polyproteins, pp1a and pp1b, that are further proteolytically processed into 16 non-structural proteins (NSPs) to form a replication–transcription complex (RTC) and several structural proteins that are required for virus replication, alteration of host defense mechanisms and the formation of new virus particles. ACE2: angiotensin-converting enzyme 2; TMPRSS2/11/13: transmembrane serine protease 2, 11 and 13; +ssRNA: positive strand RNA; pp1a and pp1(a)b: two viral polyproteins encoded by ORF1a and ORF1b, respectively; PL^pro^: SARS-CoV-2 papain-like protease; M^pro^: SARS- CoV-2 major protease; pink and green arrows: PL^pro^ and M^pro^ cleavage sites.

The analogy with SARS-CoV also helped to quickly describe the molecular mechanism of SARS-CoV-2 invasion into cells of the human respiratory system (Figure 3). Shortly after the first COVID-19 outbreak, it was already known that binding of the Spike (S) coronavirus protein to host angiotensin-converting enzyme 2 (ACE2) allows the virus to enter infected cells, but this requires previous proteolytic processing of viral S protein by the TMPRSS2 and/or endosomal papain-like cathepsins L/B [62,63]. SARS-CoV-2 uses TMPRSS2 and its related proteases (TMPRSS11/13) for S protein priming when infecting human lung cells and camostat mesylate, a small molecule inhibitor of TMPRSS2, blocks SARS-CoV-2 infection [62]. Despite its instability in vivo, more recent pharmacological analyses confirmed camostat mesylate as a potential treatment option for COVID-19, because its metabolites, 4-(4-guanidinobenzoyloxy) phenylacetic acid (GBPA) and 4-guanidoninobenzoic acid (GBA), still work as active-site inhibitors and are nearly equal in suppressing authentic SARS-CoV-2 infection in cells derived from human airway epithelia [63,64]. Although the therapeutic benefit of camostat mesylate was already observed in a retrospective analysis of critically ill patients with COVID-19 admitted to the intensive care unit (ICU) of Al Ain Hospital, Abu Dhabi, United Arabs Emirates in March 2020 [65], the recent outcomes of an investigator-initiated trial in patients hospitalized with COVID-19 do not confirm camostat mesylate as an effective treatment for hospitalized COVID-19 patients [66]. However, an effect of higher doses in the early phase of COVID-19, lowering the risk of disease progression, was not excluded. In the meantime, other TMPRSS2 inhibitors with improved potency against COVID-19 have appeared. Among them is Nafamostat mesylate, a broad-spectrum serine protease inhibitor, which is FDA approved and a frequently used drug, e.g., as an anticoagulant during hemodialysis. Nafamostat displayed higher efficiency in blocking SARS-CoV-2 cell entry and in the infection of human lung cells compared to camostat mesylate [67]. Recently, a novel class of ketobenzothiazole TMPRSS2 inhibitors with significantly improved activity over Camostat and Nafamostat have been described [68]. The IC50 of the lead compound, MM3122, against recombinant TMPRSS2 is in the subnanomolar range and this also applies to the EC50 in blocking SARS-CoV-2 host cell entry into human lung epithelial cells. Moreover, the compound has excellent metabolic stability, safety and pharmacokinetics in mice, with a half-life of 8.6 h in plasma and 7.5 h in lung tissue. These characteristics make it suitable for the evaluation of in vivo efficacy and a promising drug candidate for COVID-19 treatment.

## 6. “Tailored” Inhibitors for Better Bioavailability and Reduced Toxicity

The initial disillusionment with protease-based chemotherapy originated from the intolerable toxicity of protease inhibitors and their limited bioavailability. Although the clinical introduction of HIV-PIs and their constant use as a part of a highly effective antiretroviral therapy (HAART) has resulted in a dramatic decline in HIV-related morbidity and mortality, reflected in the prolonged lifespan of HIV patients, it often resulted in serious side effects such as insulin resistance leading to type 2 diabetes [12]. Although some adverse effects from early designs of these inhibitors disappeared after the introduction of next-generation compounds, most of the proposed inhibitors did not pass the early stages of clinical trials and the off-target adverse drug effects have remained a major concern in protease-based drug design [69].

While previous chapters outlined novel protease targets for malaria and COVID-19, here we will assess the potential drawbacks of small molecule inhibitors designed to inhibit these enzymes. “Tailoring” molecular structures of novel compounds can certainly lead to more effective and lower toxicity drugs. The danger of off-target effects can be initially limited by the characterization of the exact biological roles of target proteases and by correct estimations of unwanted cross-activity of inhibitory compounds. Molecular structuring has now been assisted by the accessibility of multiple DNA and protein sequence datasets, the availability of reliable functional genomic tools and rapid advances in protein structure observation, prediction and design. The adverse effects of inhibitors of the HIV-PIs result from the lack of specificity for the HIV proteases, which is a more distantly related enzyme to human cathepsin D, E and pepsin enzymes, than malarial plasmepsins. This highlights the potential toxicological risks caused by inadequate selectivity for plasmepsins in the treatment of malaria, as reported recently [70]. This work clearly shows that plasmepsin IX/X inhibitors can be used for selective targeting of the malarial parasite, but novel compounds should be assayed for inhibitory activity against the main human proteases, particularly cathepsins D and E, and risks should be further assessed in animal studies.

A good example of tailoring effective inhibitors that widen the therapeutic window by reducing host toxicity are malarial proteasome inhibitors based on carmaphycin B, a natural proteasome inhibitor consisting of four structural subunits: leucine epoxyketone (P1), methioninesulfone (P2), valine (P3) and hexanoic acid (P4) moieties [71]. Studies evaluating 20 synthetic analogs of carmaphycin B scaffold conclusively demonstrated that toxicity of these molecules to human cells can be dramatically reduced, while the antimicrobial activity against *P. falciparum* is still comparable to the parental compound [32]. The leading compound, tagged as analog18, has a 100-fold wider therapeutic window than carmaphycin B and consists of substitutions of D-valine for L-valine, and norleucine for methionine sulfone. In vitro evolution in the yeast model *S. cerevisiae*, biochemical assays and molecular modeling studies confirm that this activity is due to specific inhibition of the β5 subunit of the proteasome. Altogether, these findings create a strong premise for minimal toxicity and side effects of new antimalarials based on proteasome inhibition. Additionally, the emergence and rapid spreading of novel SARS-CoV-2 across the globe enhanced the use of in silico tools such as integrated machine-learning-based drug-repurposing strategies. Although the outputs of such studies await further experimental confirmation, compounds resulting from these analyses have much better in silico safety profiles when compared to existing antivirals inhibiting SARS-CoV-2 proteases [72].

## 7. Conclusions

This review focuses on the three most significant examples of successful progress in determining enzymatic targets for novel protease-based drug therapies. Inhibitors of these proteolytic enzymes represent novel drug candidates for the treatment of the two most affecting infectious diseases of human populations to date. The overall goal of this review is not to provide a comprehensive insight into the topic, but rather it should serve as a brief yet timely summary for readers working on identification and characterization of molecular targets in the development of novel therapeutic strategies for various other infectious diseases. We believe that the proteolytic targets described here for malaria and COVID-19 provide sufficient evidence to return the spotlight to protease-based drug development as an established approach to innovative therapies. This especially resonates in these times of the persisting COVID-19 pandemic, when research teams and pharmaceutical companies are designing and testing novel inhibitors based on the protein structure and physicochemical properties of the active site of SARS-CoV-2 and associated host proteases. Some of these compounds have already entered clinical trials (e.g., the phase 1 study of oral antiviral clinical candidate PF-07321332 by Pfizer, the inhibitor of the SARS-CoV-2 major protease M^pro^). This further confirms rational design of protease inhibitors as an established platform for drug development, applicable to such challenges as the ongoing COVID-19 outbreak. In addition, monoclonal antibodies (mAbs) should be considered for future applications because they have already been demonstrated to inhibit pathogenic proteases with desired selectivity [73] and thus represent an alternative therapeutic agent to low-molecular-weight protease inhibitors.
